# Trends of denosumab-related publications in web of science

**DOI:** 10.1097/MD.0000000000032784

**Published:** 2023-01-27

**Authors:** Xiaohong Jiang, Tianyu Xie, Wenyu Feng, Zhaojie Qin, Shijie Liao, Yun Liu, Shenglin Lu, Mingwei He, Qingjun Wei

**Affiliations:** a Department of Trauma Orthopedic and Hand Surgery, The First Affiliated Hospital of Guangxi Medical University, Nanning, China; b Department of Orthopedic, The Affiliated Minzu Hospital of Guangxi Medical University, Nanning, China; c Department of Spinal Bone Disease, The First Affiliated Hospital of Guangxi Medical University, Nanning, China; d Collaborative Innovation Centre f Regenerative Medicine and Medical Bioresource Development and Application Co-constructed by the Province and Ministry, Guangxi Medical University, Nanning, Guangxi, China; e Guangxi Key Laboratory of Regenerative Medicine, Research Centre for Regenerative Medicine, Guangxi Medical University, Nanning, China.

**Keywords:** bibliometric analysis, bone resorption, clinical trials, denosumab, publications

## Abstract

Denosumab is a human monoclonal antibody that targets nuclear factor-kappa B ligand and is highly effective in blocking bone resorption. Bibliometrics can intuitively show the research development process, research status, research hotspots and development trend of a certain topic for researchers. This study assessed the course of research and development for denosumab in terms of publications over the past 2 decades. Web of Science databases were searched to identify publications related to research on denosumab from January 1, 2005 to December 31, 2022. The VOS Viewer software (version 1.6.17) and Bibliometrix package in R (version 4.1.3) were used in this study. There were 5119 denosumab-related publications during this period. The total number of citations of denosumab-related publications reached 94917. The most articles were published in the field of Endocrinology Metabolism. As an international language, English remains the most popular language for writing papers. Five of the top ten institutions originated in the USA. Through the VOS Viewer analysis, we found that the relationships between Amgen Inc. with its collaborations were grouped into 4 clusters, the USA was the mainland for research and development on denosumab, closely collaborating with many other countries, such as Canada, Japan, England, and China. Wagman RB from USA was the most prolific author with 119 publications. The journal with the most publications was Osteoporosis International (481 publications). The most cited article was “Denosumab for Prevention of Fractures in Postmenopausal Women with Osteoporosis” with 2053 citations. The clinical trial comprised 6 of the 10 most frequently cited publications, and the rest consisted of reviews. The most frequent keywords for publications since January 1, 2014 were “prevention” and “management,” indicating that a number of prevention and management measures have been developed to regulate the use of denosumab in treating osteoporosis. Our research provided a comprehensive review of denosumab-related publications, suggesting that the development of denosumab is a long process and numerous clinical trials have been conducted before applications in clinical settings.

## 1. Introduction

Osteoporosis is a most common bone disease of low bone mass and increased bone fragility, usually leading to a consequent increase in fracture risk.^[1,2]^ It is mainly caused by osteoclast-induced bone resorption. Osteoclasts are the primary bone-resorbing cells derived from the monocyte/macrophage lineage.^[3]^ In bone tissues, osteoclast genesis is mediated by a receptor activator of the nuclear factor-kappa B ligand (RANKL), a protein crucial for the differentiation of osteoclasts involved in bone resorption.^[4]^ The realization that RANKL is the final cytokine involved in the resorption process quickly led to lines of therapy. Denosumab is a human monoclonal antibody that targets RANKL and is proven to be highly effective in blocking bone resorption. It successfully reduces fractures and is now one of the therapeutic options for osteoporosis treatment.^[5]^

Bibliometrics is an interdisciplinary science that uses mathematical and statistical methods to quantitatively analyze all knowledge carriers. It is a comprehensive knowledge system that integrates mathematics, statistics and literature, and pays attention to quantification. Literature information such as authors, vocabulary size, and number of documents is effective information for finding research frontiers and foci.^[6]^ Bibliometrics conducts clustering and other operations on literature information through software. After several rounds of iteration (data analysis, data cleaning, and reanalysis), it can intuitively show the research development process, research status, research hotspots and development trend of a topic for researchers, and provide decision-making information for researchers. Before our study was conducted, although there had been many studies on denosumab, no bibliometric articles were published.

In this study, we assessed all research publications on denosumab from the Web of Science database from January 1, 2005 to December 31, 2022. We utilized Bibliometrics to analyze the research and development course of denosumab for publication trends, journals, institutions, and citations. We look forward to providing insightful information about the research and development for denosumab.

## 2. Methods

We performed a computerized Bibliometric analysis from January 1, 2005 to December 31, 2022 for papers about “denosumab” retrieved from the Web of Science database. In detail, to identify all publications, we used “denosumab” as the TOPIC and refined the timespan to “January 1, 2005 to December 31” (indexes = science citation index expanded, current chemical reaction expanded, and index chemicals). Data were collected in December 2022. The search and data extraction were independently conducted by 2 of our investigators. The titles and abstracts of potentially eligible publications were reviewed and the unrelated were excluded. Mismatched choices were resolved involving a third independent researcher and the final decision was made by consensus among researchers.

All extracted publications from the Web of Science starting from January 1, 2005 to December 31, 2022 were finally retrieved and analyzed. The impact factor of each publication was obtained from the 2022 journal citation report (JCR) database, and the citations of each included publication were retrieved from the Web of Science tools. (JCR region) in the JCR was based on the journal impact factor in the year, and the journals were classified into 4 regions: Q1, Q2, Q3, and Q4, in that order: Q1 > Q2 > Q3 > Q4 (Q: Quartile in category). The journal ranking and impact factor might measure the journal influence and research worth. For Bibliometric analysis, we applied the inherent functions of the Web of Science to detect the trends and characteristics, such as countries/regions of origin, institutions, citation counts, h index, journals, and so on. We also identified the top 10 cited institutions, articles, and authors. VOS Viewer software (version 1.6.17, https://www.vosviewer.com/)^[7]^ and Bibliometrix package in R (version 4.1.3, https://cran.r-project.org/)^[8]^ were used for data and text-mining, analysis, and visualization. GraphPad Prism (version 8.0) and Adobe Illustrator (version 15.0.0) were used for generating figures.

## 3. Results

### 3.1. Publication trends

**Figure 1.**
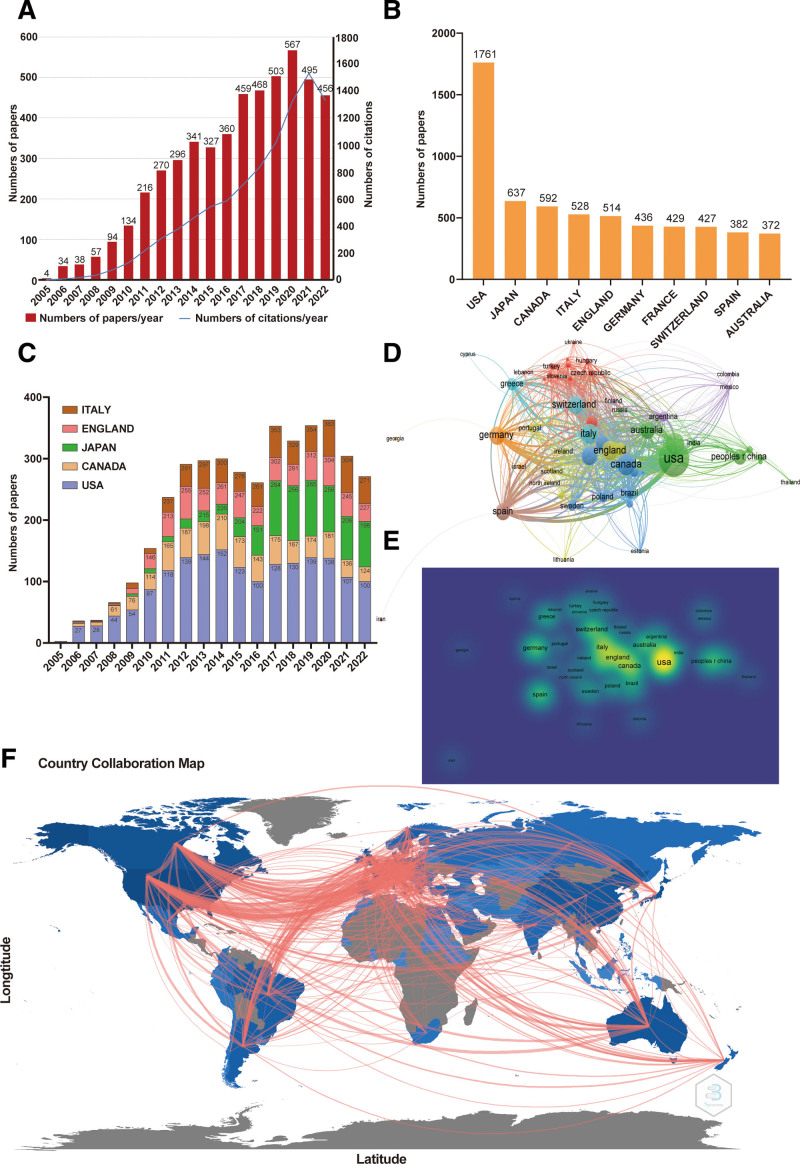
Characteristics of denosumab-related publications. (A) Annual global number and citations of denosumab publications. (B) Top 10 countries/regions of denosumab-related publications. (C) Time histogram of denosumab-related publications from the top 5 countries. (D–E) Collaborations among different countries analyzed by VOS Viewer. (F) Collaborations among different countries analyzed by Bibliometrix package in R.

We identified a total of 5119 qualified articles published during the period January 1, 2005 to December 31, 2022, starting with just 4 publications in 2005 and reaching 456 publications in 2022. The total number of citations was 94917, and the number of citations per year increased from 1 in 2005 to 13,315 in 2022 (Fig. 1A). Eighty-nine countries/regions had research and publications on denosumab, among which the USA ranked the first with 1661 articles, followed by Japan with 637 and Canada with 592 (Fig. 1B). The number of publications of the top 5 countries did not show an increasing trend in the past 5 years (Fig. 1C). Moreover, we found that the articles from the USA largely determined the trend of global publications (Fig. 1C). By VOS Viewer (Fig. 1D–E) and Bibliometrix package in R analysis (Fig. 1F), we found that the USA was the mainland for research and development on denosumab, closely collaborating with many other countries, such as Canada, Japan, England, and China.

### 3.2. Language distributions

**Figure 2.**
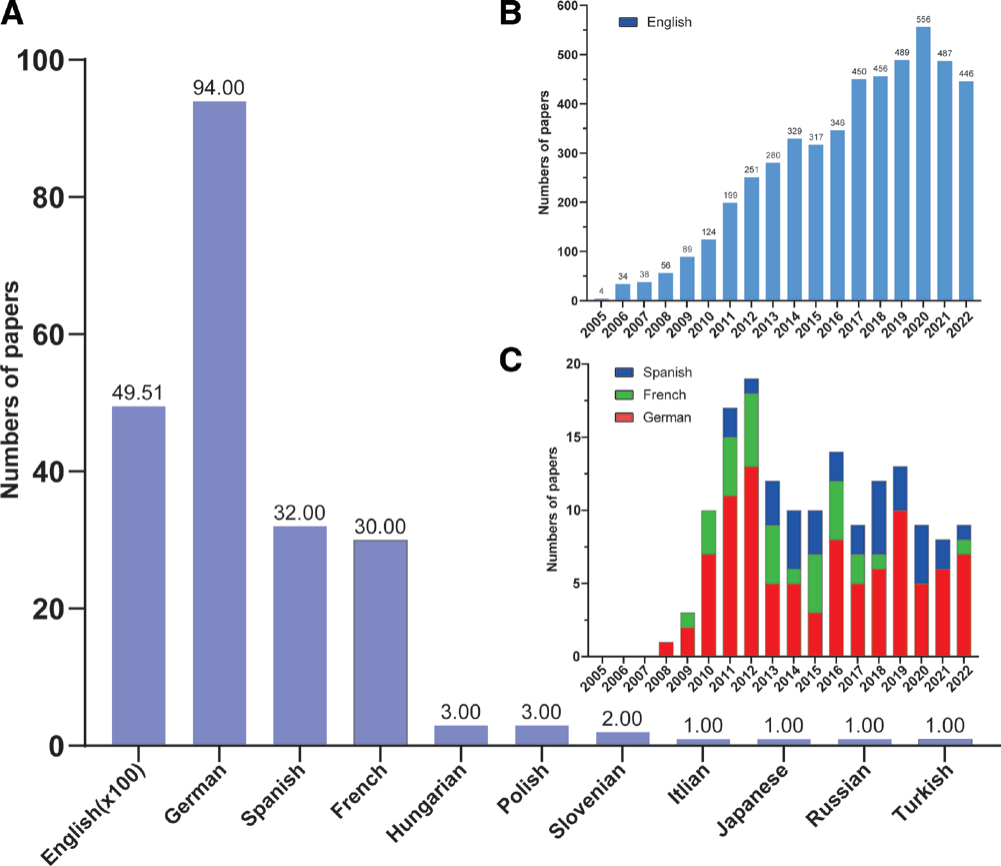
Most productive languages on denosumab. (A) Most published languages for research on denosumab. (B) Annual global number of denosumab publications published in English. (C) Time histogram of denosumab-related publications published in Spanish, French, and German.

Eleven languages used in denosumab-related publications were listed in our data (Fig. 2A). As expected, English ranked first with 4951 publications, followed by German with 94. Interestingly, Spanish and French ranked a third and 4th place with the publication number of 32 and 30. Furthermore, we identified 4 English publications in 2005 and 446 in 2022, which suggested a gradually increasing trend from 2005 to 2020 (Fig. 2B). The number of German (second), Spanish (third) and French (4th) publications, however, did not show an increasing trend in the past 5 years (Fig. 2C).

### 3.3. Leading productive institutions

**Figure 3.**
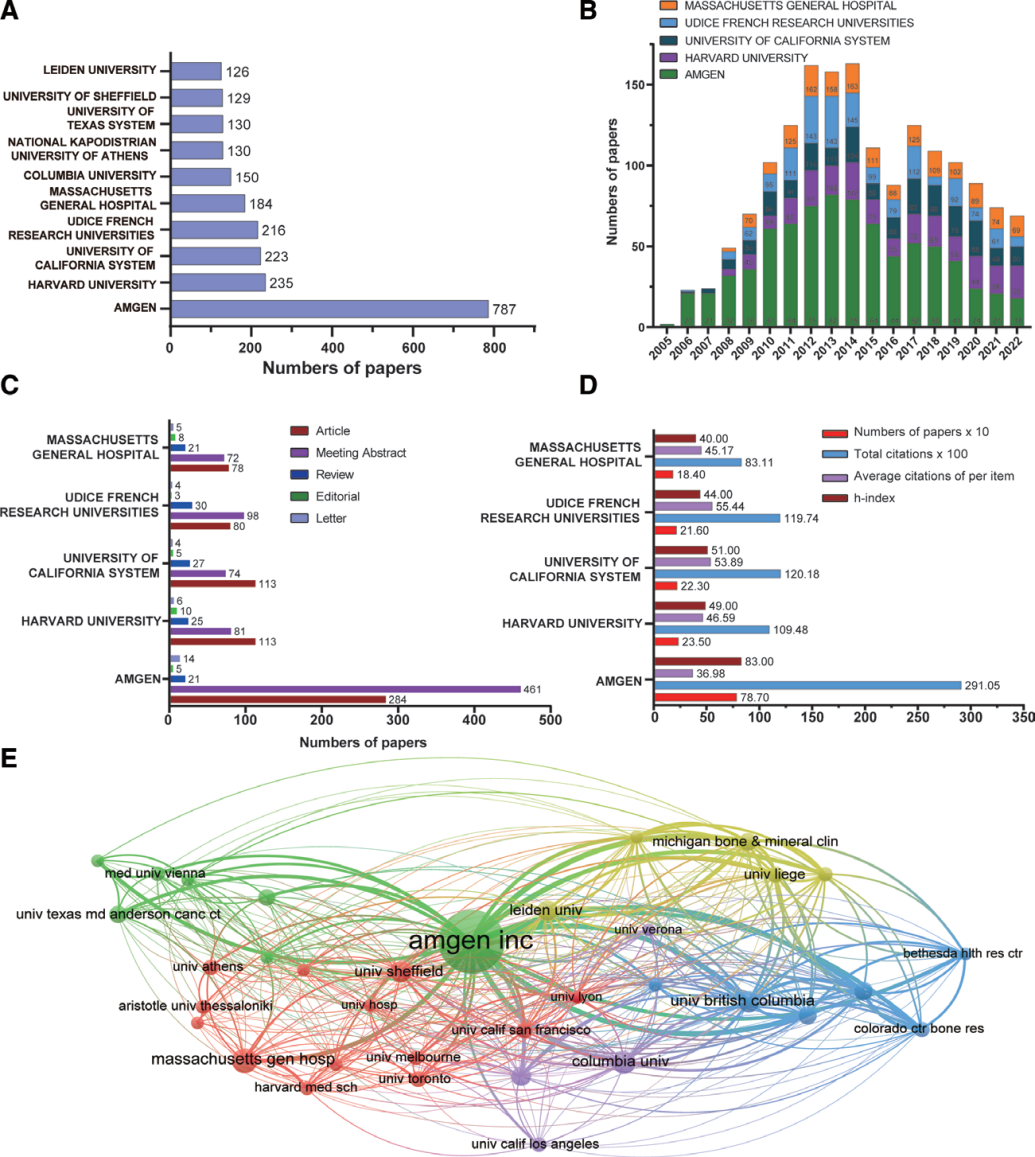
Most productive institutions and their collaborations. (A) Top 10institutions for research on denosumab. (B) Time histogram of denosumab-related publications from the top 5institutions. (C) Distribution of research data on denosumab. (D) Five most active first institutionsand their citation reports. (E) Collaborations among different institutions analyzed by VOS Viewer.

The top 10 productive institutions are listed in Figure 3A. Specifically, Amgen Inc. published 787 articles, followed by Harvard University with 235, and the University of California system with 223. Interestingly, of these top 10 most productive institutions, half were from the USA, including Amgen Inc., Harvard University, University of California system, Massachusetts General Hospital, and Columbia University. Interestingly, we found that the scale of articles in Amgen Inc. determined the trends of American publications (Fig. 3B). We also found that the main types and categories of publications of Amgen Inc. were “Meeting Abstract,” followed by “Article” (Fig. 3C). Notably, the 787 publications from Amgen Inc. ranked first with 29,105 citations (36.98 citations per article), for an h index number of 83 (Fig. 3D). In terms of the quality of articles, there was no significant gap between American institutions and those in other countries. Through the VOS Viewer analysis, we found that the relationships between Amgen Inc. with its collaborations were grouped into 4 clusters (Fig. 3E), suggesting that collaboration connections among these institutions were relatively concentrated.

### 3.4. Distribution of authors

**Figure 4.**
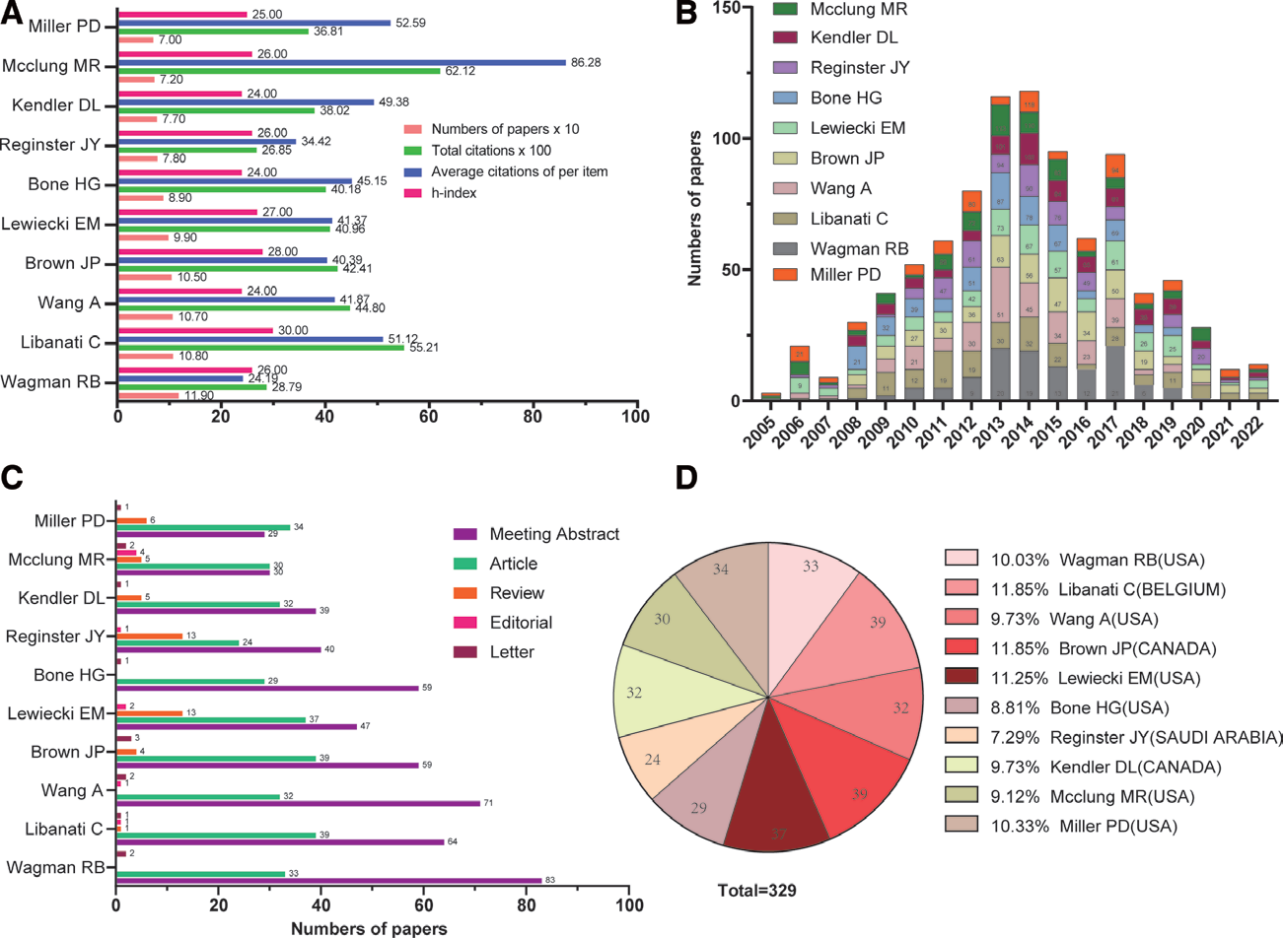
Most productive authors in research on denosumab. (A) Top 10 first authors and their citation reports. (B) Denosumab-related publications from top 10 first authors. (C) Distribution of research data on denosumab from top 10 first authors. (D) Plot of proportion of “articles” from the top 10 first authors of “article” from these top 10 first authors.

We identified a total of 16,769 researchers who participated in publishing the denosumab publications. The top 10 most productive paper-published authors were Wagman RB (119), Libanati C (108), Wang A (107), Brown JP (105), Lewiecki EM (99), Bone HG (89), Reginster JY (78), Kendler DL (77), Mcclung MR (72), and Miller PD (70) (Fig. 4A–B). Interestingly, we found that the top 3 most paper-published authors (Wagman RB, Libanati C, and Wang A) once all worked at Amgen Inc. However, the publications of Mcclung MR from Oregon Osteoporosis Ctr., Portland, who had never worked at Amgen Inc. before, occupied the highest number of citations (6212). Similarly, we found that the main types and categories of publications of these top 10 most productive paper-published authors were “Meeting Abstract,” followed by “Article” (Fig. 4C). Brown JP from Laval University (Canada) and Libanati C from UCB Pharma SA (Belgium) participated in the greatest number of published “Article” about denosumab both with 39, followed by Lewiecki EM from New Mexico Clinical Research & Osteoporosis Center (USA) with 37 (Fig. 4D and Table S1, Supplemental Digital Content, http://links.lww.com/MD/I385).

### 3.5. Research types and categories

**Figure 5.**
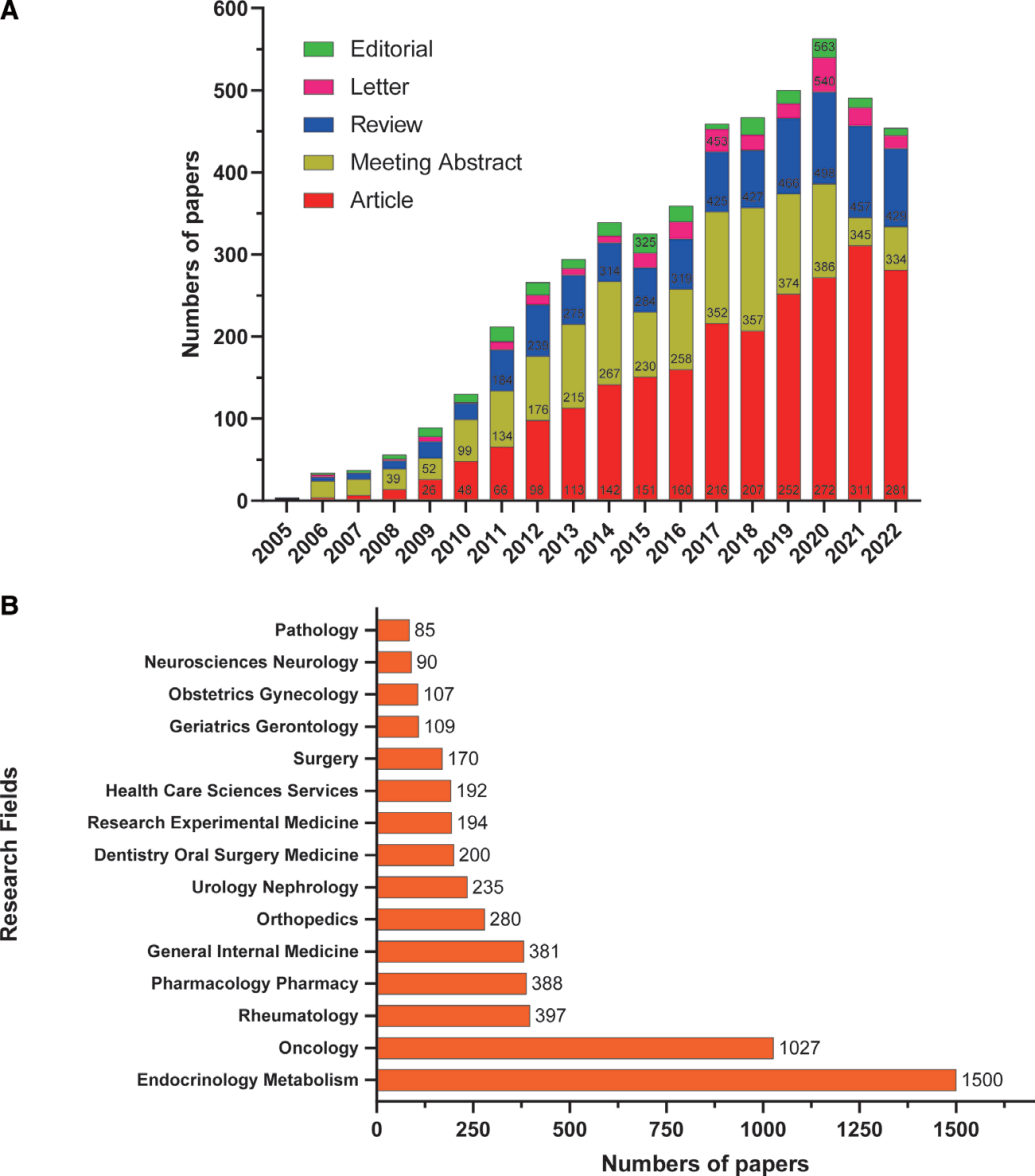
Research types and categories of denosumab-related publications. (A) Time histogram of the worldwide distribution of publication types. (B) Top 10 research fields of the publications.

The types and categories of denosumab-related publications are shown in Figure 5. The main types of denosumab-related publications were “Article,” “Meeting Abstract,” “Review,” “Letter,” and “Editorial.” “Article,” and “Review” both showed an increasing trend since January 1, 2005, while “Meeting Abstract” decreased gradually in these 3 years, especially in 2021 (Fig. 5A). Additionally, there were 71 research categories related to denosumab globally in our data. Endocrinology Metabolism was the main research type and category with1, 500 publications, followed by Oncology with 1027, and Rheumatology with 397 (Fig. 5B). The top 10 journals of denosumab-related publications are shown in Table 1. *Osteoporosis International* ranked first with 481 published articles, followed by *Journal of Bone and Mineral Research* with 320 and *Bone* with 144. According to the JCR (2022), *Journal of Bone and Mineral Research, Journal of Clinical Oncology, Annals of the Rheumatic Diseases, Journal of Clinical Endocrinology and Metabolism, Annals of Oncology, Arthritis Rheumatology*, and *Value in Health* were classified as Q1, while *Osteoporosis International, Bone* and *Archives of Osteoporosis* were classified as Q2.

**Table 1:** Top 10 journals of the publications.

Rank	Journal	Number of papers	Total citations	Average citations	IF (JCR 2022)	h-index
1	Osteoporosis international	481	4774	9.93	5.071 (Q2)	39
2	Journal of bone and mineral research	320	8490	26.53	6.39 (Q1)	48
3	Bone	144	3255	22.6	4.626 (Q2)	30
4	Journal of clinical oncology	135	4345	32.19	50.739 (Q1)	22
5	Annals of the rheumatic diseases	115	384	3.34,	28.003 (Q1)	5
6	Journal of clinical endocrinology metabolism	98	3904	39.84	6.134 (Q1)	34
7	Annals of oncology	65	1371	21.09	51.769 (Q1)	12
8	Arthritis rheumatology	64	419	6.55	15.483 (Q1)	4
9	Value in health	59	177	3	5.156 (Q1)	4
10	Archives of osteoporosis	58	463	7.98	2.879 (Q2)	12

JCR = journal citation report.

### 3.6. Top 10 most cited articles

The top 10 most cited articles (6 clinical trials and 4 reviews) for denosumab are listed in Table 2. The most frequently cited article was a clinical trial article, “Denosumab for Prevention of Fractures in Postmenopausal Women with Osteoporosis” by Cummings Steven R. et al^[9]^ in *New England Journal of Medicine*. This is followed by a review, “Osteoporosis: Now and the future” by Rachner Tilman D. et al^[10]^ in *Lancet*. The third most frequently cited article was a clinical trial, “Denosumab versus zoledronic acid for treatment of bone metastases in men with castration-resistant prostate cancer: A randomized, double-blind study” by Fizazi Karim. et al^[11]^ in *Lancet*, it is worth mentioning that this article had been retracted for unknown reasons on December 1, 2011 (https://www.auajournals.org/doi/10.1016/j.juro.2011.08.108).

**Table 2:** The top 10 most cited publications in denosumab research.

	Title	First author	Journal	Date	Total citation	Impact factor	Quartile in category	Article type
1	Denosumab for prevention of fractures in postmenopausal women with osteoporosis	Cummings, Steven R.	New England journal of medicine	Aug 20, 2009	2053	91.253	Q1	Clinical trial
2	Osteoporosis: now and the future	Rachner, Tilman D.	Lancet	Apr 9, 2011	1522	79.323	Q1	Review
3	Denosumab vs zoledronic acid for treatment of bone metastases in men with castration-resistant prostate cancer: a randomized, double-blind study	Fizazi, Karim.	Lancet	Mar 5, 2011	1326	79.323	Q1	Clinical trial
4	Denosumab compared with zoledronic acid for the treatment of bone metastases in patients with advanced breast cancer: a randomized, double-blind study	Stopeck, Alison T.	Journal of clinical oncology	Dec 10, 2010	1029	44.544	Q1	Clinical trial
5	EAU guidelines on prostate cancer. Part ii: treatment of advanced, relapsing, and castration-resistant prostate cancer	Heidenreich, Axel	European urology	Feb 2014	1017	20.096	Q1	Review
6	EAU-ESTRO-SIOG guidelines on prostate cancer. Part ii: treatment of relapsing, metastatic, and castration-resistant prostate cancer	Cornford, Philip	European Urology	Apr 2017	991	20.096	Q1	Review
7	Denosumab in postmenopausal women with low bone mineral density	McClung, MR	New England journal of medicine	Feb 23, 2006	852	91.253	Q1	Clinical trial
8	Atypical subtrochanteric and diaphyseal femoral fractures: second report of a task force of the American society for bone and mineral research	Shane, Elizabeth	Journal of bone and mineral research	Jan 2014	841	6.741	Q1	Review
9	Randomized, double-blind study of denosumab vs zoledronic acid in the treatment of bone metastases in patients with advanced cancer (excluding breast and prostate cancer) or multiple myeloma	Henry, David H.	Journal of clinical oncology	Mar 20, 2011	775	44.544	Q1	Clinical trial
10	Denosumab in men receiving androgen-deprivation therapy for prostate cancer	Smith, Matthew R.	New England journal of medicine	Aug 20, 2009	749	91.253	Q1	Clinical trial

### 3.7. Keywords analysis

**Figure 6.**
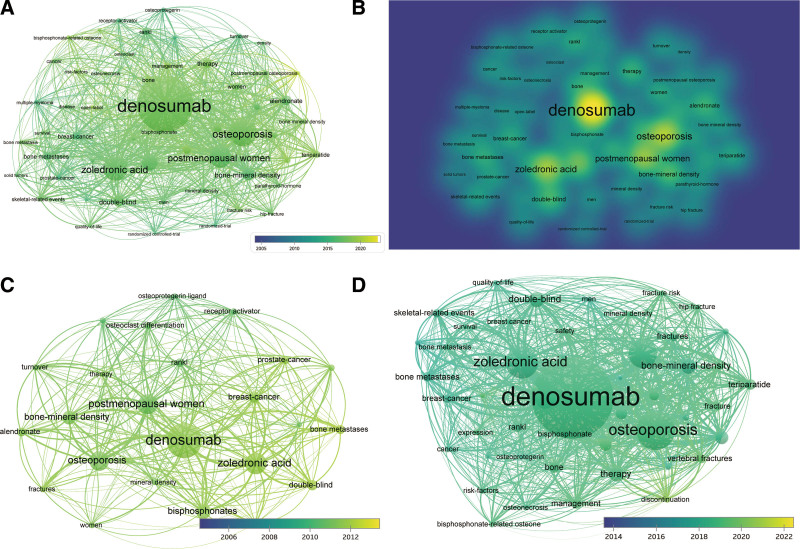
Keyword analysis for publications on denosumab by VOS Viewer. (A) Co-occurring keywords during the period January 1, 2005 to December 31, 2022. (B) Density map show the heat of keywords during the period January 1, 2005 to December 31, 2022. (C) Co-occurring keywords in publications from January 1, 2005 to December 31, 2013. (D) Co-occurring keywords in publications from January 1, 2014 to December 31, 2022.

To detect the trends and directions of denosumab-related publications, we performed VOS Viewer analysis on the distributions of co-occurring keywords, which met the minimum number of occurrences of a keyword no < 50 times. The results of the analysis presented that the keywords were divided into 3 categories: “indications (purple),” “medications (green),” and “treatments (yellow)” (Fig. 6A–B). In the “indications” group, the most popular keywords were “breast-cancer,” “prostate-cancer,” and “bone metastases.” In the “medications” group, the most popular keywords were “denosumab,” “zoledronicacid,” “alendronate,” “postmenopausal women,” and “double-blind.” In the “treatments” group, the most popular keywords were “denosumab,” “therapy,” “management,” and “prevention.” In the stage from January 1, 2005 to December 31, 2013, the more popular topics were on clinical treatment, such as “zoledronicacid,” “double-blind,”“osteoporosis,” and “bisphosphonates” appeared. With the progression of denosumab, more common keywords appeared. For example, hot topics such as “prevention” and “management” appeared more frequently from January 1, 2014 to December 31, 2022, indicating that the hot topics changed dynamically over time (Fig. 6C–D).

## 4. Discussion

Increasing evidences have confirmed the efficacy of denosumab on the treatment of bone loss, including postmenopausal osteoporosis, primary bone tumor, and bone metastases.^[12–14]^ In this study, we conducted a Bibliometric analysis of denosumab-related publications from January 1, 2005 to December 31, 2022. We found that denosumab-related publications rapidly increased by nearly a hundredfold since January 1, 2005. The USA dominated in denosumab research and development, closely followed by Canada and Japan. In our data, most publications originated from companies and institutions in the USA, such as Amgen Inc., Harvard University, and University of California system. These organizations worked with a variety of academic institutions and hospitals to promote clinical trials and use of denosumab. There was also a considerable gap between other major countries and the USA in the number of publications. Specifically, we found the scale of the USA articles largely determined the trend of global publications. Note that the USA occupied the highest number of articles published in all countries in 2014. After that, however, the number of articles published in the USA fell to the lowest level in 2016 and then gradually increased to another peak in 2019. In comparison, the number of articles published in Japan gradually increased since 2009 and only slightly decreased until 2020.

According to the JCR (2022), most journals that published denosumab-related publications were classified as Q1, indicating a high research and impact value. Most journals that published denosumab-related publications were in the field of osteoporosis, especially clinical research. With the increasing number of clinical trials, researchers have expanded from osteoporosis to primary bone tumor, such as osteosarcoma and giant cell tumor of bone.^[12,13]^ It has been reported that denosumab could lead to a positive increase in bone mineral density and a low rate of fractures and is safe and effective in patients with severe renal impairment.^[14]^ However, in view of correlating to rare adverse events, like osteonecrosis of the jaw and atypical femoral fractures,^[15]^ many drug candidates will need to be investigated. Thus, dominant countries like the USA, Canada, and China should strengthen their support in completing high-quality clinical trials.

In the top 10 most cited denosumab-related articles, 6 were clinical researches and 4 were reviews. Nowadays, the concept of translational medicine is continually being strengthened because it bridges the vital gap between basic research and clinical applications. Our data also confirmed that the characteristics of denosumab-related publications are according to the perception of translational medicine.

Likewise, our findings indicated the changes in hot topics over the past years. For instance, the keywords related to “zoledronicacid,” “double-blind,” “osteoporosis,” and “bisphosphonates” were popular from 2005 to 2013, whereas the recent keywords associated with clinical applications, such as “prevention” and “management,” appeared from 2014 to 2022. This result indicates that with the increasing evidences of the clinical effectiveness of denosumab, numerous prevention and management measures have been developed to regulate the use of denosumab in the treatment of osteoporosis.^[16,17]^Thus, it is not surprising that “prevention” and “management” appeared more commonly during the latest 8 years.

Unavoidably, there are limitations in our current research. For example, we only searched publications in the Web of Science database, which may result in the exclusion of other types of publications and, in turn, incomplete data collection. Citations and h-index reports for some publications may have been delayed as well, leading to systematic bias in this research.

## 5. Conclusion

Our research provided a comprehensive review of denosumab-related publications, suggesting that the development of denosumab is a long process and numerous clinical trials have been conducted before applications in clinical settings.

## Acknowledgments

We would like to thank all the members participated in this study.

## Author contributions

**Conceptualization:** Qingjun Wei.

**Data curation:** Xiaohong Jiang, Yun Liu.

**Formal analysis:** Tianyu Xie, Mingwei He.

**Investigation:** Shijie Liao.

**Methodology:** Shijie Liao, Shenglin Lu.

**Software:** Wenyu Feng, Zhaojie Qin.

**Supervision:** Qingjun Wei.

**Validation:** Zhaojie Qin.

**Visualization:** Wenyu Feng, Zhaojie Qin.

**Writing – original draft:** Xiaohong Jiang.

**Writing – review & editing:** Yun Liu, Shenglin Lu, Mingwei He.

## Supplementary Material
