# Supplementary material for: Trends of denosumab-related publications in web of science
Source: Medicine (Baltimore). 2023 Jan 27;102(4):e32784. doi: 10.1097/MD.0000000000032784 (PMC9876013; doi:10.1097/MD.0000000000032784)
Supplement: Supplementary file 1 [file medi-102-e32784-s001.pdf]

Supplementary table. Top 10 authors of the publications.

Rank	Author	Number of papers	Percentage (%)	Country	Institution
1	Wagman RB	33	10.03	USA	Myovant Sci Inc
2	Libanati C	39	11.85	BELGIUM	UCB Pharma SA
3	Wang A	32	9.73	USA	Amgen
4	Brown JP	39	11.85	CANADA	Laval University
5	Lewiecki EM	37	11.25	USA	New Mexico Clinical Research & Osteoporosis Center
6	Bone HG	29	8.81	USA	Michigan Bone & Mineral Clin
7	Reginster JY	24	7.29	SAUDI ARABIA	King Saud University
8	Kendler DL	32	9.73	CANADA	University of British Columbia
9	Mcclung MR	30	9.12	USA	Oregon Osteoporosis Center
10	Miller PD	34	10.33	USA	Colorado Ctr Bone Hlth
